# Turnover of Sex Chromosomes in the Stickleback Fishes (Gasterosteidae)

**DOI:** 10.1371/journal.pgen.1000391

**Published:** 2009-02-20

**Authors:** Joseph A. Ross, James R. Urton, Jessica Boland, Michael D. Shapiro, Catherine L. Peichel

**Affiliations:** 1 Division of Human Biology, Fred Hutchinson Cancer Research Center, Seattle, Washington, United States of America; 2 Graduate Program in Molecular and Cellular Biology, University of Washington, Seattle, Washington, United States of America; 3 Summer Undergraduate Research Program, Fred Hutchinson Cancer Research Center, Seattle, Washington, United States of America; 4 Department of Biology, University of Utah, Salt Lake City, Utah, United States of America; University of Oxford, United Kingdom

## Abstract

Diverse sex-chromosome systems are found in vertebrates, particularly in teleost fishes, where different systems can be found in closely related species. Several mechanisms have been proposed for the rapid turnover of sex chromosomes, including the transposition of an existing sex-determination gene, the appearance of a new sex-determination gene on an autosome, and fusions between sex chromosomes and autosomes. To better understand these evolutionary transitions, a detailed comparison of sex chromosomes between closely related species is essential. Here, we used genetic mapping and molecular cytogenetics to characterize the sex-chromosome systems of multiple stickleback species (Gasterosteidae). Previously, we demonstrated that male threespine stickleback fish (*Gasterosteus aculeatus*) have a heteromorphic XY pair corresponding to linkage group (LG) 19. In this study, we found that the ninespine stickleback (*Pungitius pungitius*) has a heteromorphic XY pair corresponding to LG12. In black-spotted stickleback (*G. wheatlandi*) males, one copy of LG12 has fused to the LG19-derived Y chromosome, giving rise to an X_1_X_2_Y sex-determination system. In contrast, neither LG12 nor LG19 is linked to sex in two other species: the brook stickleback (*Culaea inconstans*) and the fourspine stickleback (*Apeltes quadracus*). However, we confirmed the existence of a previously reported heteromorphic ZW sex-chromosome pair in the fourspine stickleback. The sex-chromosome diversity that we have uncovered in sticklebacks provides a rich comparative resource for understanding the mechanisms that underlie the rapid turnover of sex-chromosome systems.

## Author Summary

Sex chromosomes have independently evolved many times in animals, plants, and fungi. Although some sex chromosomes have been maintained across different species for over a hundred million years, there are other systems in which sex chromosomes appear to turn over within a few million years. Because sex determination is such a fundamental biological process, this rapid turnover of sex chromosomes is puzzling. Theoretical work has attempted to identify the mechanisms that might underlie the transitions between sex-chromosome systems. However, there are few empirical data to test these hypotheses. In this study, we have uncovered an unanticipated diversity of sex-chromosome systems in the stickleback fishes (Gasterosteidae). We have found that each stickleback species examined has a unique sex-chromosome system, with evidence for two independent XY systems, a derived Y-autosome fusion that has created an X_1_X_2_Y neo-sex chromosome, and a ZW system. Thus, stickleback fishes are excellent model species for investigating the mechanisms that lead to rapid turnover of sex chromosomes.

## Introduction

Genetic sex determination (GSD) is prevalent in vertebrates and is often accompanied by the presence of a heteromorphic chromosome pair in one sex. Birds and snakes have a ZW heteromorphic pair, where the W sex chromosome is female-limited; however, neither the bird nor the snake sex determination locus has been identified (reviewed in [1]). Most mammals have an XY heteromorphic pair [2], and the male-limited Y sex chromosome bears *SRY*, a male-determining gene [3]–[5] that is found in all but a handful of mammals [6]–[10]. However, this broad conservation of sex-chromosome systems across large taxonomic groups is not universal in vertebrates. Both simple (XY and ZW) and complex (polygenic) forms of GSD, as well as environmental sex determination (ESD), are seen in teleost fish, lizards, turtles and amphibians [1]. Even closely related species within a genus might have different sex-determination systems. For example, the only other known vertebrate sex-determining gene, *DMY* in the medaka fish *Oryzias latipes* [11],[12], is not found in many closely related *Oryzias* species [13],[14].

Comparative studies of sex-chromosome systems have supported the assertion that sex-chromosome pairs were autosomes prior to the acquisition of a sex-determination locus [15]. Sex-chromosome heteromorphy initially arises as a consequence of selection for a loss of recombination between linked sex-determining loci [16]. Once recombination is suppressed, intrachromosomal inversions and deletions and mobile sequence elements tend to accumulate in the nonrecombining region of the Y or W chromosome [2], [17]–[20]. These physical changes to the sex chromosome result in heteromorphy seen in metaphase chromosome spreads, although it is not possible to state *a priori* whether the hemizygous sex chromosome will be the larger or smaller chromosome of a heteromorphic pair [21].

Theoretical studies have suggested that a further reduction of recombination on a sex chromosome might be favored when genes with alleles of sexually-antagonistic effect are linked to a sex-determination locus (*SEX*) [22]. For example, an allele that increases male fitness and reduces female fitness in an XY system benefits from absolute linkage with the male-determining *SEX* locus. Selection for linkage of sexually-antagonistic genes to *SEX* might also explain the rapid turnover of sex-determination loci and sex chromosomes between closely related species [23]. Several mechanisms could bring about this rapid turnover, including the appearance of a novel *SEX* locus on an autosome [15], the transposition of a *SEX* locus between chromosomes in different lineages [24], or fusions of an existing sex chromosome with an autosome [25]. Investigation of the process of sex-chromosome turnover requires detailed molecular, genetic, cytogenetic and phylogenetic analyses of sex determination systems that differ between closely related species.

Teleost fishes are a particularly useful group to explore turnover of sex chromosome systems because different sex-determination mechanisms exist in closely related species [26]–[29]. For example, there is evidence for the evolution of a novel *SEX* locus in *Oryzias* [28] and transposition of the existing *SEX* locus in salmonids [24]. Furthermore, both XY and ZW GSD systems have been identified in species of *Oryzias* [28],[30],[31], *Xiphophorus* [32], and tilapiine cichlids [29],[33],[34]. Thus, teleost fishes also provide the opportunity to ask whether distinct forms of GSD found in closely related species have interconverted or evolved independently.

**Figure 1:**
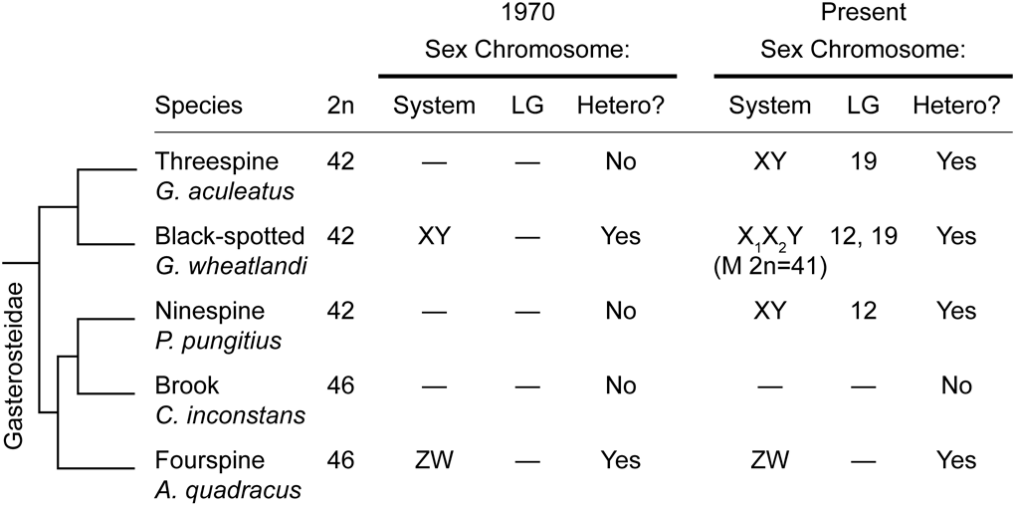
Phylogeny of North American stickleback fishes (Gasterosteidae). A summary of the 1970 cytogenetic data [35] and the present data (incorporating [38]–[40]). The stickleback phylogeny is based on [61],[62]. The common and scientific name of each species is given, along with the diploid number of chromosomes, type of sex determination system, linkage group to which *SEX* maps, and presence of a heteromorphic sex chromosome pair. In *G. wheatlandi*, females have the diploid chromosome number originally reported (2n = 42); the number of chromosomes in males (M) is 41. — indicates that no data are available.

Among fishes, the sticklebacks (Gasterosteidae) provide a particularly interesting system in which to investigate the evolution of sex determination and sex chromosomes. The first cytogenetic survey in this family reported the presence of a heteromorphic XY pair in the black-spotted stickleback (*Gasterosteus wheatlandi*) and a heteromorphic ZW pair in the fourspine stickleback (*Apeltes quadracus*) [35]. In the same study, evidence of a heteromorphic pair was not seen in the threespine stickleback (*G. aculeatus*). The findings of this 1970 study, along with phylogenetic relationships between the stickleback species, are summarized in Figure 1.

Although later studies [36],[37] also did not find evidence of a heteromorphic sex-chromosome pair in *G. aculeatus*, genetic mapping subsequently identified the presence of XY genetic sex determination on linkage group (LG) 19 in this species [38]. Using fluorescence *in situ* hybridization (FISH), we have recently demonstrated that there is a heteromorphic XY pair corresponding to LG19 in *G. aculeatus* [39]. Genetic mapping has now demonstrated that the sex-determination locus in the ninespine stickleback (*Pungitius pungitius*) maps to LG12, which is distinct from the *G. aculeatus* sex chromosome LG19 [40]. Taken together, these data suggest that different sex-determination systems and sex chromosomes have evolved within the stickleback clade. Combined with the recent development of genetic and genomic resources for both *G. aculeatus* and *P. pungitius* [40]–[43], these small teleost fish are an excellent system in which to study the evolution of sex chromosomes and GSD.

To systematically characterize the relationships between the sex-determination mechanisms and sex-chromosome systems in the stickleback family, we genetically mapped sex-determination loci and searched for heteromorphic sex-chromosome pairs using FISH in the North American stickleback species: *G. wheatlandi*, *P. pungitius*, the brook stickleback (*Culaea inconstans*), and *A. quadracus*. Using FISH in *P. pungitius*, we identified a heteromorphic XY pair corresponding to LG12, where the sex-determination locus has been mapped in this species [40]. We confirmed the presence of a heteromorphic pair in *G. wheatlandi*, although we find that males of this species have a diploid chromosome number of 41, not 42 as previously reported [35]. Genetic mapping and molecular cytogenetics demonstrate that the *G. wheatlandi* Y chromosome consists of a fusion between LG12 and LG19, resulting in an X_1_X_2_Y sex-chromosome system. However, neither LG12 nor LG19 is associated with a sex-determination locus or a heteromorphic sex-chromosome pair in *C. inconstans* or *A. quadracus*. Consistent with previous work [35], we find that *A. quadracus* has a heteromorphic ZW pair, while *C. inconstans* has no heteromorphic sex-chromosome pair. The results we report here, summarized in Figure 1, demonstrate the remarkable diversity of genetic mechanisms and chromosomal systems of sex determination that can be present within a clade of teleost fish that diverged approximately twenty million years ago (MYA) [44].

## Results

### *SEX* Is Linked to Both LG12 and LG19 in *G. wheatlandi*

Master sex-determination loci map to different Y chromosomes in *G. aculeatus* (LG19) and *P. pungitius* (LG12) [38],[40]. The relationships between linkage groups in these two species were established by including markers of known genomic locations derived from *G. aculeatus* in the *P. pungitius* linkage map [40]. Furthermore, the sequences of the *P. pungitius* microsatellite markers used for map construction were BLASTed against the *G. aculeatus* genome (http://www.ensembl.org/Gasterosteus_aculeatus/Info/Index), and the positions of 92% (156/169) were unambiguously identified [40]. The nomenclature for the *P. pungitius* linkage groups corresponds to the *G. aculeatus* nomenclature, and LG12 and LG19 represent distinct chromosomes in these two species [40].

To determine whether markers from either sex-chromosome pair (LG12 or LG19) are linked to *SEX* in *G. wheatlandi*, we genotyped the 80 progeny (41 females and 39 males) of three *G. wheatlandi* crosses with LG12 and LG19 markers derived from both *G. aculeatus* and *P. pungitius* (Table S1). Five markers from LG19 and six markers from LG12 were heterozygous in at least one of the parents of the three crosses (Table S1). For the nine markers that were heterozygous in a male parent, there was perfect concordance between the marker genotype inherited from the father and the sex phenotype of the progeny (Table S2), demonstrating that *G. wheatlandi* males are the heterogametic (XY) sex and that markers from both LG12 and LG19 are sex-linked in *G. wheatlandi*. For all five LG19 markers, the Y-linked allele is a null allele (i.e. no PCR product is amplified), while none of the Y-linked alleles of LG12 markers are null.

**Figure 2:**
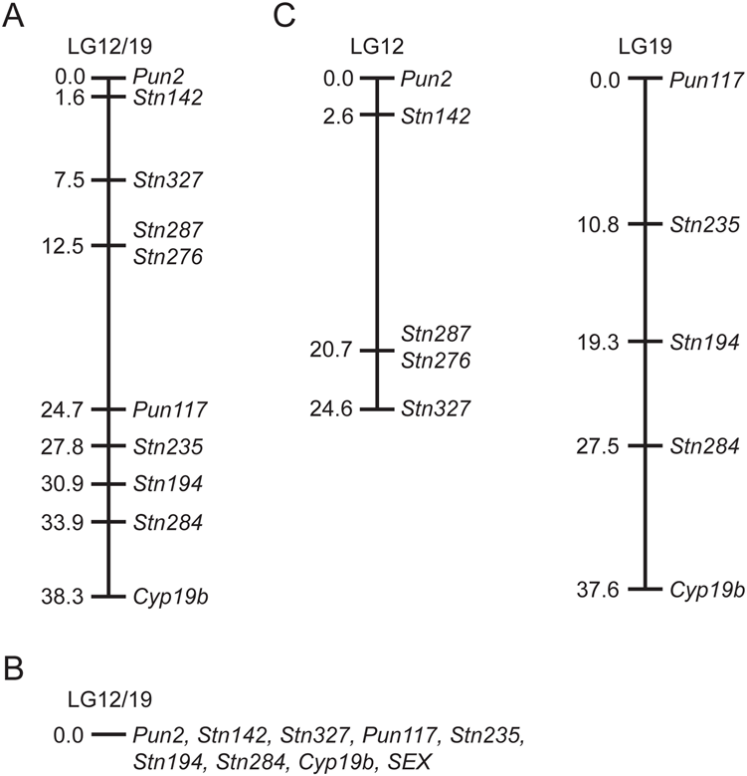
Genetic linkage maps of the *G. wheatlandi* sex-chromosome pair. (A) combined male and female meiotic data. (B) male meiotic data only. (C) female meiotic data only.

To further explore the relationship between LG12 and LG19 markers in these *G. wheatlandi* crosses, the complete genotypes of the ten markers informative in all three crosses were used to create a linkage map. Using a stringent LOD score of 10.0, all ten markers were found in a single linkage group (Figure 2A). When only the male meiotic data were used to create a linkage map, all markers were completely linked to each other and to *SEX* (Figure 2B). However, when only the female meiotic data were used to create a linkage map, two independent linkage groups representing LG12 and LG19 were found (Figure 2C).

### Cytogenetic Evidence of a Fusion between LG12 and LG19 in *G. wheatlandi* Males

**Figure 3:**
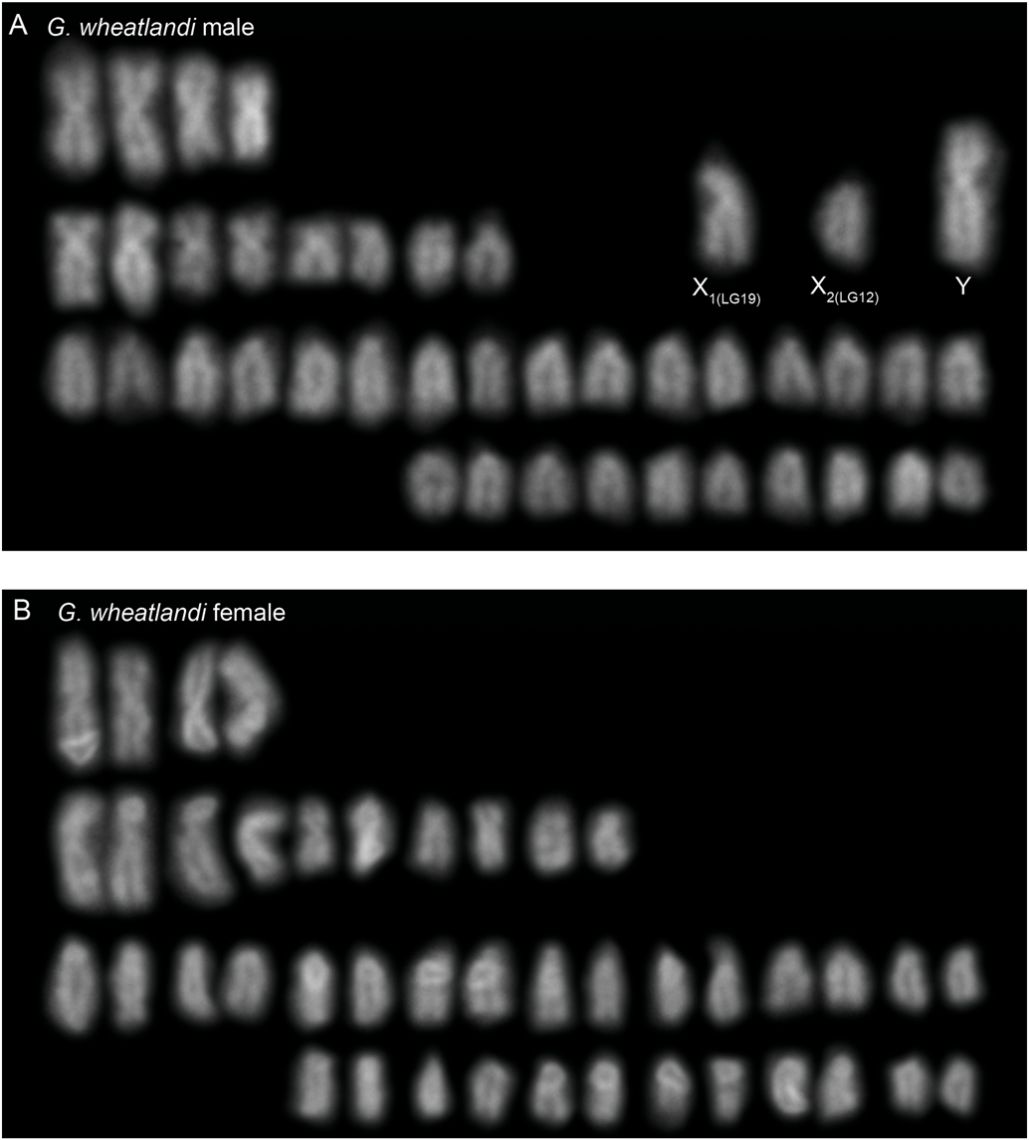
Karyograms of *G. wheatlandi*. (A) *G. wheatlandi* male. The Y chromosome and two presumed X chromosomes are indicated. (B) *G. wheatlandi* female.

Our genetic mapping data suggest that one chromosome 12 and one chromosome 19 might be fused in male, but not female, *G. wheatlandi*. Consistent with these results, a karyogram made from a male *G. wheatlandi* metaphase spread (Figure 3A) contained 41 chromosomes (19 pairs and three unpaired). The presence of 41 chromosomes in male somatic tissue was seen in multiple metaphase spreads from multiple individuals from two different populations (Table 1; Materials and Methods). The three unpaired chromosomes consist of a large submetacentric, a medium submetacentric, and a medium acrocentric chromosome. By contrast, the female karyogram comprises 21 chromosome pairs (Figure 3B). Absence of the large submetacentric chromosome from the female karyogram defines it as the Y (Figure 3A).

**Table 1:** Karyotype data for stickleback fishes.

Species	Sex	# individuals	# metaphases analyzed	Mode 2n	% metaphases with mode 2n	2n range
*G. wheatlandi*	Male	4	56	41	87%	36–41
	Female	3	20	42	85%	40–42
*P. pungitius*	Male	1	16	42	69%	39–42
	Female	5	33	42	85%	39–43
*C. inconstans*	Male	3	40	46	78%	41–47
	Female	4	40	46	65%	40–47
*A. quadracus*	Male	10	17	46	59%	41–47
	Female	11	49	46	85%	35–47

For both sexes of each species, column 3 gives the number of individuals from whom diploid chromosome number counts were obtained; the total number of metaphase spreads analyzed is given in column 4. The mode diploid chromosome number, which we present as the true diploid chromosome number, is in column 5. The percentage of total metaphases analyzed having the mode chromosome number is shown in column 6, and the range of chromosome counts for all metaphase spreads is given in column 7. The identification of fewer chromosomes than the mode in a metaphase spread is likely due to chromosomes overlapping in a metaphase spread and being counted as one, or to nearby cellular debris obscuring chromosomes. The identification of more chromosomes than the mode in a metaphase spread could be due to chromosomes having tortuous morphology or uneven DAPI staining and being counted as two or more chromosomes, or to the presence of chromosomes from neighboring nuclei near the metaphase spread.

**Figure 4 pgen-1000391-g004:**
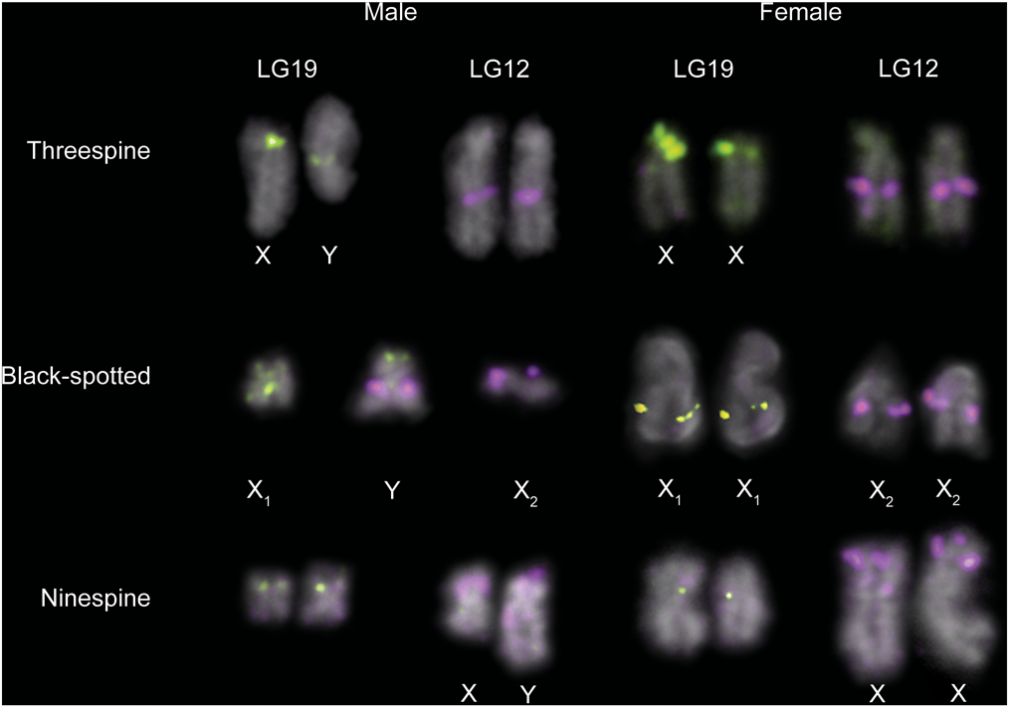
Fluorescence *in situ* hybridization analyses of LG12 and LG19 in *G. aculeatus*, *G. wheatlandi*, and *P. pungitius*. The LG19 probe CH213-180J08 (*Wt1a*) probe was used in all experiments except for the *G. wheatlandi* male, where the LG19 probe CH213-035N15 (*Stn303*) was used. The LG19 probes are green and the LG12 probe (CH213-140B10) is purple. For each sex of each species, chromosomes from a single metaphase spread are shown. The *G. aculeatus* LG19 sex chromosome pair is heteromorphic in males, while LG12 is not. The *P. pungitius* male LG12 pair is heteromorphic, while LG19 is not. In *G. wheatlandi* males, one distinct copy each of LG12 and LG19 is present, while probes to both LGs hybridize to the two arms of a large, submetacentric, male-specific chromosome (the Y). LG12 and LG19 are homomorphic in females of all three species. The chromosome sizes cannot be compared between sexes or species because they were obtained from different metaphases.

**Figure 5:**
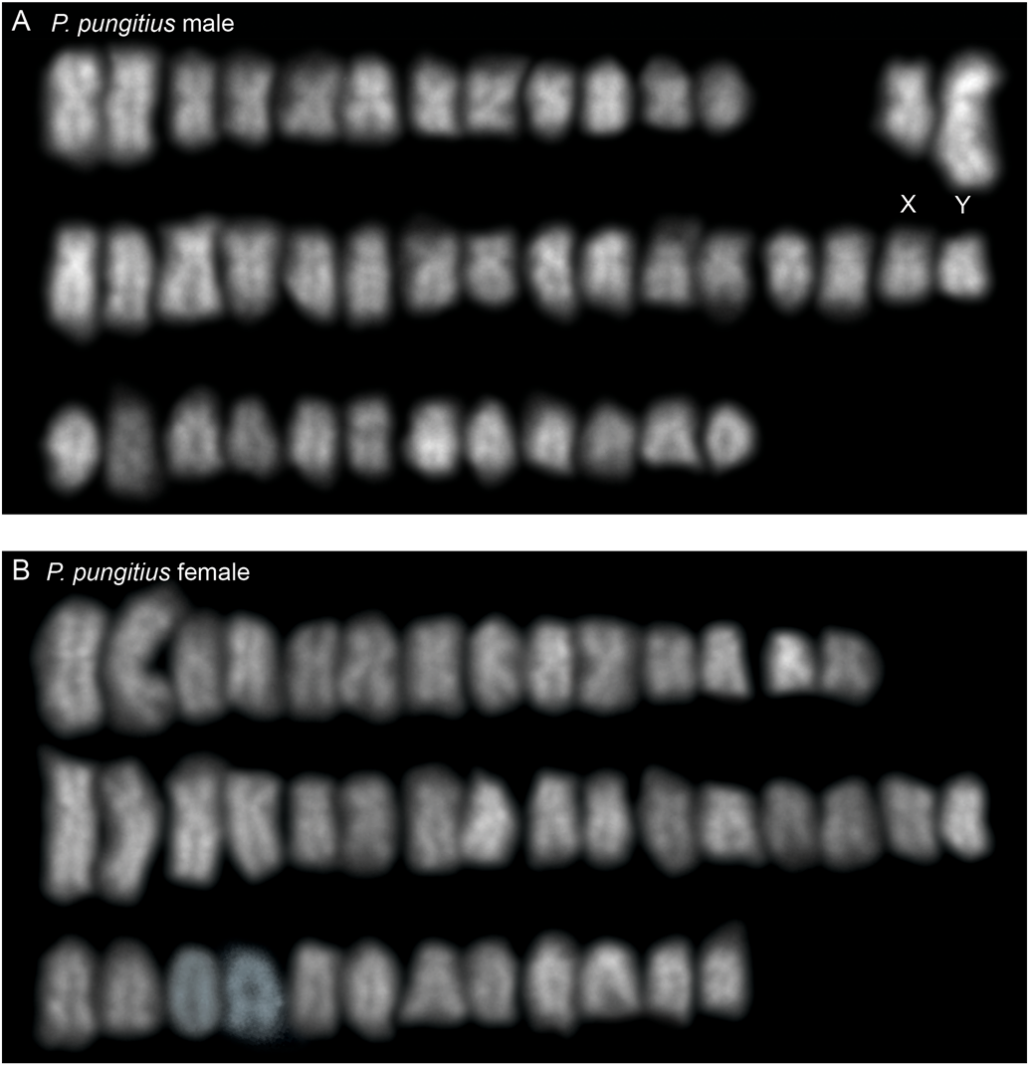
Karyograms of *P. pungitius*. (A) *P. pungitius* male. The presumed X and Y chromosomes are indicated. (B) *P. pungitius* female.

To examine the relationship between LG12 and LG19 in *G. wheatlandi*, *G. aculeatus* and *P. pungitius*, we hybridized LG12 and LG19 FISH probes to metaphase spreads from females and males of all three species. In females of all three species, the LG12 and LG19 pairs appear homomorphic (Figure 4). We had previously demonstrated that the *G. aculeatus* LG19 is heteromorphic in males [39]; here we demonstrate that LG12 is homomorphic in males (Figure 4). There is a heteromorphic pair in the male *P. pungitius* karyogram (Figure 5A) that is absent from the female karyogram (Figure 5B). However, we found that LG12, and not LG19, comprises the heteromorphic pair in male *P. pungitius* (Figure 4). Because both copies of chromosome 12 (the X chromosome) in female *P. pungitius* are metacentric, the metacentric chromosome 12 in *P. pungitius* males is the X, and the submetacentric chromosome 12, which appears larger than the X, is the Y chromosome (Figure 4; Figure 5A).

In male *G. wheatlandi* metaphase spreads, both the LG12 probe and the LG19 probe for the *Stn303* locus [39] hybridized to a large unpaired submetacentric chromosome, which is the Y chromosome (Figure 3A; Figure 4). The LG12 probe also hybridized to the medium unpaired acrocentric chromosome, while the LG19 probe hybridized to the medium unpaired submetacentric chromosome (Figure 4). In female *G. wheatlandi* metaphase spreads, the LG12 probe hybridized to a pair of acrocentric chromosomes, and the LG19 probe hybridized to a pair of submetacentric chromosomes (Figure 4). Based on a comparison of chromosome morphologies across species, we define the submetacentric chromosome 19 to be X_1_ and the acrocentric chromosome 12 to be X_2_ for *G. wheatlandi* males (Figure 3A; Figure 4).

**Figure 6:**
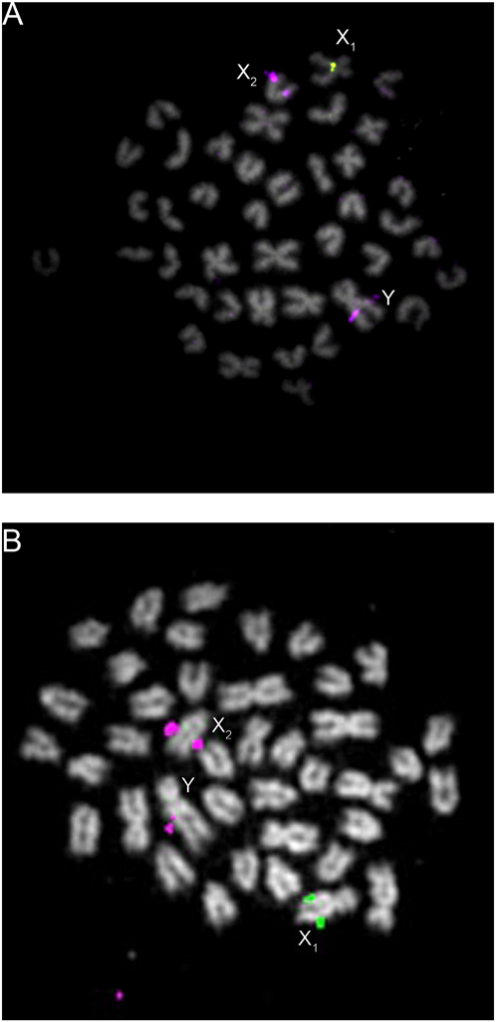
Certain LG19 FISH probes do not hybridize to the *G. wheatlandi* Y. In male *G. wheatlandi* metaphase spreads, the LG19 probes (A) CH213-180J08 (*Wt1a*) and (B) CH213-101E08 (*Idh*) in green do not hybridize to the Y, while the LG12 probe (CH213-140B10) in purple does hybridize to the Y. Both LG19 probes hybridize to the X_1_ (LG19).

To further assess the physical relationship between the *G. wheatlandi* and *G. aculeatus* Y chromosomes, we used two additional LG19 FISH probes, one from the *Idh* locus and one from the *Wt1a* locus [39]. Each probe hybridized to a chromosome pair in female *G. wheatlandi*. However, each probe only hybridized to a single chromosome in males and did not hybridize to the large submetacentric Y (Figure 6), indicating that loci present on the *G. aculeatus* Y might be deleted from the *G. wheatlandi* Y. These data are consistent with the finding of null Y-linked alleles for all five LG19 markers used to genotype the *G. wheatlandi* mapping crosses.

### *SEX* Is Not Linked to LG12 or LG19 in *C. inconstans* or *A. quadracus*

We next asked whether *SEX*-linked markers from LG12 or LG19 were associated with a single locus controlling male or female sexual development in two additional stickleback species. We genotyped a single *C. inconstans* cross and a single *A. quadracus* cross and found no such associations (Table S1). Seven LG12 markers (4 from *G. aculeatus* and 3 from *P. pungitius*) were informative in our *A. quadracus* cross, yet none were linked to *SEX* (Table S1). Similarly, two LG19 markers, *Stn194* and *Pun117*, were linked to *SEX* in *G. aculeatus* but not in *A. quadracus* (Table S1). Although very few LG12 and LG19 markers were informative in our *C. inconstans* cross, neither the LG12 marker *Pun234* nor the four LG19 markers, *Stn186*, *Cyp19b*, *Pun168*, and *Pun268*, were linked to *SEX* in *C. inconstans* (Table S1).

To determine whether any *G. aculeatus* or *P. pungitius* genetic markers are linked to a sex-determination locus in these species, we genotyped the *C. inconstans* cross and the *A. quadracus* cross with all available *G. aculeatus* and *P. pungitius* markers [40],[41]. There was no evidence for an association between any marker and sex phenotype in either species. However, many *G. aculeatus* and *P. pungitius* markers either failed to work in *C. inconstans* or *A. quadracus*, or were not heterozygous in the parents of the crosses (Table S3). Therefore, we were unable to test markers from all *G. aculeatus* and *P. pungitius* linkage groups.

### *A. quadracus* Has a ZW Sex Chromosome System

**Figure 7:**
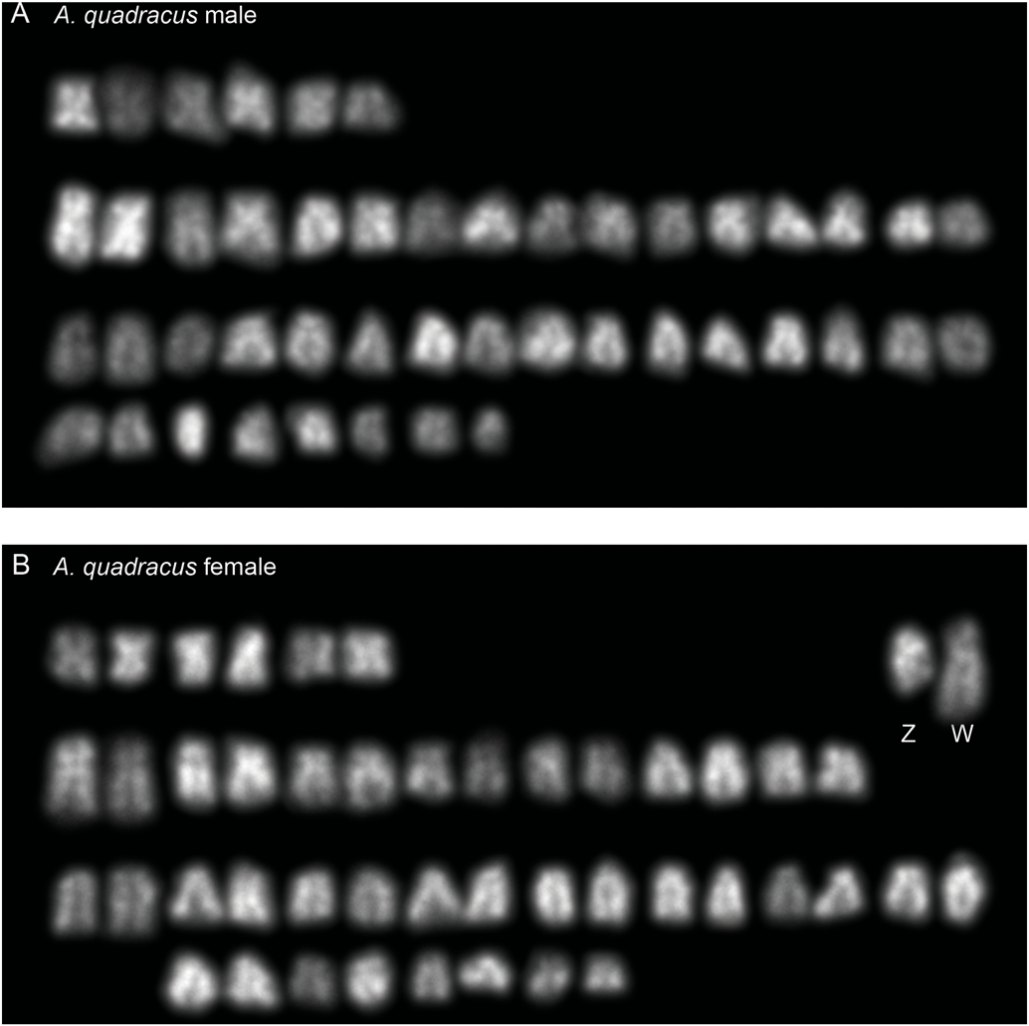
Karyograms of *A. quadracus*. (A) *A. quadracus* male. (B) *A. quadracus* female, with the W and presumed Z chromosomes labeled.

We hybridized LG12 and LG19 FISH probes to metaphase spreads from *A. quadracus* and *C. inconstans* males and females. Consistent with the genetic mapping data in these species, neither of the chromosome pairs identified by hybridization with LG12 and LG19 probes were heteromorphic in either sex of either species (Figure S1). There was no evidence for obvious heteromorphy of any chromosome pair in *C. inconstans* males or females (Figure S2). By contrast, there is a heteromorphic chromosome pair in the female *A. quadracus* karyogram, in which the female-limited W chromosome appears larger than the Z at metaphase (Figure 7).

## Discussion

### Identification of Heteromorphic Sex-Chromosome Pairs in Sticklebacks

Our cytogenetic survey of the family Gasterosteidae has uncovered a diversity of sex- chromosome systems not previously identified [35]. Although initial cytogenetic surveys of stickleback fish did not find a heteromorphic XY pair in *P. pungitius* [35],[36], genetic mapping has shown that *P. pungitius* have an XY pair corresponding to LG12 [40]. Here, we used FISH to demonstrate that chromosome 12 is a heteromorphic pair in *P. pungitius* males (Figure 4). Although we only examined karyotypes from a single Canadian population of *P. pungitius*, our results are very similar to those obtained in a recent cytogenetic study of two Polish populations, in which a heteromorphic XY pair was also identified [45].

While our *G. wheatlandi* male karyogram supports the prior report of a heteromorphic XY pair, the same study also reported a male diploid chromosome number of 42 [35]. Our karyogram shows that male *G. wheatlandi* have a 2n = 41 karyotype, while females are 2n = 42 (Figure 3). It is unlikely that presence of an odd diploid chromosome number in males is due to experimental artifact, as this result was obtained in multiple metaphase spreads from multiple individuals obtained from natural populations in Canada and Massachusetts (Table 1; Materials and Methods). Furthermore, the genetic mapping data supporting the physical linkage between LG12 and LG19 was obtained using a third *G. wheatlandi* population from Maine. Although we have not found evidence for intraspecific polymorphism in the stickleback sex chromosomes studied here, we cannot rule this out as a possible reason for the discrepancy between the current study and previous work [35]. However, we believe that the higher resolution of the molecular cytogenetic techniques used here explain our findings of additional heteromorphic sex-chromosome systems in the Gasterosteidae.

### An X_1_X_2_Y Sex Chromosome System in *G. wheatlandi*

Genetic and cytogenetic evidence support the conclusion that a fusion between the *G. aculeatus* Y chromosome (Y_LG19_) and an autosome (LG12) created a neo-Y chromosome in *G. wheatlandi* males. This fusion created an X_1_X_2_Y sex chromosome system, which explains the odd diploid chromosome number in *G. wheatlandi* males. Our data suggest that the X_1_X_2_Y system in *G. wheatlandi* is derived from the *G. aculeatus* Y chromosome, rather than the *P. pungitius* Y chromosome. All available phylogenies support a closer relationship between *G. aculeatus* and *G. wheatlandi* than between *G. wheatlandi* and *P. pungitius* (Figure 1). Our cytogenetic and FISH data also support a closer relationship between the *G. aculeatus* and *G. wheatlandi* karyotypes: LG12 is acrocentric in *G. aculeatus* and *G. wheatlandi* females, but metacentric in *P. pungitius* females, while LG19 is the submetacentric X chromosome in *G. aculeatus* and *G. wheatlandi*, but metacentric in *P. pungitius* (Figures 3–5).

Our data are consistent with other studies in teleost fish, where species with X_1_X_2_Y systems often have one less chromosome than sister taxa [46],[47]. If the *G. wheatlandi* X_1_X_2_Y male karyotype had been created by fission of an ancestral X rather than a Y-autosome fusion, we should have observed the diploid chromosome number 2n = 43 in males, rather than the observed 2n = 41 (Figure 3A). These data suggest that derived Y chromosome-autosome fusions might be the predominant source of X_1_X_2_Y sex chromosome systems in teleost fishes, although alternative mechanisms likely account for X_1_X_2_Y systems discovered in insects and mammals [48].

Our FISH data further suggest that the fusion of the ancestral Y_LG19_ chromosome and the acrocentric LG12 autosome resulted in loss of one arm of the Y_LG19_ in *G. wheatlandi* males (Figure 6). The two *G. aculeatus* LG19 FISH probes that did not hybridize to the *G. wheatlandi* Y (Figure 6) are from the q arm of the *G. aculeatus* Y (Yq) [39], while the LG19 probe that did hybridize to the *G. wheatlandi* Y (Figure 4) is from the p arm of the *G. aculeatus* Y [39]. Furthermore, the Y-linked alleles of all four *G. aculeatus* Yq markers segregated as null alleles in *G. wheatlandi*, consistent with loss of the q arm. In further support of the hypothesis that LG12 was autosomal prior to fusion with the Y, none of the LG12 markers have null alleles, suggesting that extensive degeneration has not yet occurred on the LG12-derived region of the *G. wheatlandi* Y. This is consistent with the interpretation that one copy of LG12 fused to the existing Y_LG19_ chromosome in *G. wheatlandi* males in the past 10 million years since *G. wheatlandi* and *G. aculeatus* diverged [44]. The loss of one arm of Y_LG19_ is consistent with the creation of the *G. wheatlandi* Y by an unbalanced Robertsonian translocation between a metacentric Y and an acrocentric autosome.

To more precisely map the rearrangements that have occurred between the two X chromosomes and the Y chromosome in *G. wheatlandi*, we will need a more extensive cytogenetic analysis as we have accomplished for the *G. aculeatus* X and Y [39]. However, the current data suggest that additional deletions of Y chromosome material have occurred on the *G. wheatlandi* Y relative to the *G. aculeatus* Y. No hybridization of FISH probes containing the *Wt1a* or *Idh* genes is observed on the *G. wheatlandi* Y chromosome (Figure 6), although these two probes do hybridize to the *G. aculeatus* Y chromosome [39]. In particular, the deletion of the region around the *Idh* FISH probe on the *G. wheatlandi* Y (Figure 6B) explains why a male-specific allele of this locus was not identified in our previous study [38], leading to the erroneous conclusion that the Y chromosomes of these two species were unrelated. Finally, the putative loss of an entire arm of the Y chromosome in *G. wheatlandi* males, as well as the existence of a 6 Mb deletion on the *G. aculeatus* Y chromosome [39], suggests that dosage compensation mechanisms may have evolved in sticklebacks. We plan to investigate this possibility in the future.

### The Evolutionary History of XY GSD in Sticklebacks

Our data suggest that XY sex chromosomes have been independently derived on LG19 and LG12 in the *Gasterosteus* and *Pungitius* lineages, respectively. It is possible that sex determination also arose independently in these lineages. Precedent for the independent evolution of XY sex determination in closely related species exists in the teleost clade *Oryzias* [28]. However, it is also possible that *G. aculeatus*, *G. wheatlandi*, and *P. pungitius* all share a common sex-determining locus, but that *SEX* has transposed between LG12 and LG19 in the two lineages; a similar transposition of the *SEX* locus to four different chromosomes has been seen in salmonids [24]. Finally, although we have argued that the *G. wheatlandi* Y was derived from the *G. aculeatus* Y, it is possible that sex determination arose independently in these species. In order to distinguish these possibilities, the identity of *SEX* in these three species must be determined. If all three species share a common sex-determining factor, then transposition of *SEX* between linkage groups has likely occurred; the identification of different sex-determining factors in the three species would provide evidence of the independent evolution of XY sex determination in sticklebacks.

In any of these scenarios, LG12 appears to have been selected for *SEX* linkage at least two independent times: by fusion to the Y_LG19_ chromosome in *G. wheatlandi* and by acquisition of either a transposed or newly evolved *SEX* locus in *P. pungitius*. It has been suggested that selection for linkage between autosomal genes with sexually antagonistic effects and *SEX* might drive Y-autosome fusions [25], the appearance of a new *SEX* locus on an autosome [23], or the transposition of an existing *SEX* locus to an autosome [23]. Thus, linkage between LG12 and *SEX* in both *G. wheatlandi* and *P. pungitius* suggests that LG12 might have an abundance of genes with differential fitness effects in males and females and thus be predisposed to becoming a sex chromosome. Comparative genomic analysis of the autosomal and sex-linked forms of LG12 in the different stickleback species might yield insight into the presence and types of sexually antagonistic alleles that play an important role in the evolution of sex chromosomes in sticklebacks.

### Transitions between XY and ZW Systems

In addition to multiple XY systems, we also found evidence for both XY and ZW systems in sticklebacks. Both cytogenetic and genetic data suggest that the ZW system of *A. quadracus* is not related to the XY systems of *G. aculeatus*, *G. wheatlandi* or *P. pungitius*, raising the possibility that the ZW system arose independently. However, we do not know what the ancestral sex-chromosome state is for the sticklebacks, so an accurate parsimony-based reconstruction of the evolution of XY and ZW GSD in sticklebacks is not currently possible. It will be useful to karyotype the European fifteenspine stickleback (*Spinachia spinachia*), a close relative of *A. quadracus*, to determine whether it too has 46 chromosomes and a heteromorphic ZW pair. We are currently working to identify *SEX*-linked sequences in *A. quadracus* using unbiased methods and to identify the linkage group comprising the Z and W chromosomes by FISH with *G. aculeatus* BAC probes. These studies will allow us to determine which autosome(s) gave rise to this ZW pair.

Additional efforts will focus on identifying the sex determination mechanism of *C. inconstans*. Although we have not yet identified sex-linked markers in either *C. inconstans* or *A. quadracus*, these studies have been limited by the availability of polymorphic markers. Therefore, it is still possible that there is a simple genetic sex determination mechanism in *C. inconstans*. However, it is also possible that *C. inconstans* uses ESD or complex GSD. Knowing the sex determination mechanism in *C. inconstans* might shed light on the transitions between XY and ZW systems in this family.

Transitions between XY and ZW GSD occur via indirect or direct mechanisms. For example, an interim period of ESD might facilitate an indirect transition between two forms of GSD [1]. A more direct transition between XY and ZW forms might occur, as exemplified by recent work. In tilapiine fishes, two species (*Oreochromis aureus* and *O. mossambicus*) have complex genetic sex determination in which LG1 and LG3 are both associated with sex determination loci [29],[33]. The phylogenetic positions of these species provide a direct link between two related species in which LG1 is associated with a simple XX/XY system and two other species in which LG3 is associated with a simple ZZ/ZW system [29]. A similar link exists in the platyfish (*Xiphophorus maculatus*), where some populations have W, X, Y and Z chromosomes and closely-related species have either XY or ZW GSD [32]. In the Japanese frog (*R. rugosa*), there is evidence that an existing XY sex chromosome became a ZW sex chromosome in a derived population [49]. Finally, comparative mapping of the platypus sex chromosome chain suggests that the monotreme XY GSD system might have directly evolved from a bird-like ZW system, while the therian XY system might have evolved independently [1],[10],[50],[51]. Additional genetic and genomic analyses of all stickleback species might elucidate whether the ZW and XY systems directly interconverted or were independently derived in sticklebacks.

### Conclusions

Teleost fishes are useful organisms in which to study the evolution of sex determination and sex chromosomes. In particular, several teleost fish species have X_1_X_2_Y sex chromosome systems [46], [47], [52]–[57]. While a benefit of fusion of sex chromosomes to autosomes has been suggested [25], to our knowledge this is the first report of the evolution of an X_1_X_2_Y system in which the fused Y comprises two chromosomes that are used as distinct Y chromosomes in two closely related species. X_1_X_2_Y sex chromosome systems are not reported as frequently as XY or ZW systems, which could mean that they lead to evolutionary dead ends or exist as transitional states [58]. For example, following the fusion of a Y to an autosome, the X_1_ and X_2_ chromosomes might fuse to restore diploidy. The discovery of an X_1_X_2_Y system in *G. wheatlandi*, sister species to *G. aculeatus*, for which many molecular, genetic and genomic tools have been developed [41]–[43], will facilitate further characterization of the mechanisms and evolutionary forces underlying the transition between simple XY sex chromosomes and X_1_X_2_Y neo-sex chromosomes. Furthermore, the availability of these tools in sticklebacks will also be important to improve our understanding of the transition between male and female heterogamety that is evident in this group of fishes with diverse sex chromosome systems.

## Materials and Methods

### Ethics Statement

All animal work was approved by the Fred Hutchinson Cancer Research Center Institutional Animal Care and Use Committee (protocol #1575).

### Genetic Crosses

Three *G. wheatlandi* crosses were generated using males and females collected from Wells, ME in May 2003. Sperm from a single *G. wheatlandi* male was used to fertilize the eggs of a single *G. wheatlandi* female (cross 1); the sperm of a second *G. wheatlandi* male was used to fertilize the eggs of two different *G. wheatlandi* females (crosses 2 and 3). The progeny of each of the three crosses were grown in separate tanks. A single *C. inconstans* cross was generated using a female collected from Fox Holes Lake (Northwest Territories, Canada) and a male collected from Pine Lake (Wood Buffalo National Park, Alberta, Canada) in June 2005. A single *A. quadracus* cross was generated using a single female and a single male collected from Pilgrim Lake (Cape Cod National Seashore, MA) in May 2004. For all crosses, the sex of the progeny was determined by visual inspection of the gonads. DNA was prepared from the caudal fin of each individual by phenol-chloroform extraction, followed by ethanol precipitation.

### Microsatellite Genotyping

PCR genotyping with *G. aculeatus* microsatellite markers and *P. pungitius* microsatellite markers was performed as previously described [40],[41], except that the reactions were run on an ABI 3100 and the genotypes were analyzed using ABI GeneMapper 3.7 (Applied Biosystems).

### Linkage Analysis

Genetic linkage maps were created in JoinMap3.0 [59] using default parameters. Both Kruskal-Wallis tests for significant associations between genotype and sex phenotype, as well as interval mapping, were performed in MapQTL4.0 [60].

### Cytogenetic Analysis

Metaphase spreads were prepared as described [39] using *G. wheatlandi* males collected from Baie de L'Isle-Verte National Wildlife Area (Québec, Canada) in May 2003, *G. wheatlandi* males and females collected from Demarest Lloyd State Park (Dartmouth, MA) in May 2005 and May 2007, *P. pungitius* and *C. inconstans* males and females collected from Pine Lake (Wood Buffalo National Park, Alberta, Canada) in June 2007, *A. quadracus* males collected from Pilgrim Lake (Cape Cod National Seashore, MA) in May 2005, and *A. quadracus* males and females collected from Demarest Lloyd State Park (Dartmouth, MA) in May 2005 and 2007.

Fluorescence *in situ* hybridization (FISH) experiments were performed on metaphase spreads as described [39]. Bacterial artificial chromosomes (BACs) from the *G. aculeatus* CHORI-213 library [42] were used as FISH probes. Linkage group (LG) 19 probes (*Stn303*: CH213-035N15; *Idh*: CH213-101E08; and *Wt1a*: CH213-180J08) were identified previously [39]. The LG12 probe (CH213-140B10), spanning 0.35 to 0.56 Mbp on the *G. aculeatus* public genome assembly of scaffold 6 (part of the LG12 sequence), was found to contain genetic marker *Stn144* [41] at 0.51 Mbp using a computational approach [39].

## Supporting Information

Figure S1 Fluorescence *in situ* hybridization analyses of LG12 and LG19 in *C. inconstans* and *A. quadracus*. The LG19 probe (CH213-180J08) is green, and the LG12 probe (CH213-140B10) is purple. For each sex of each species, only chromosomes hybridized by probe in a single metaphase spread are shown. LG12 and LG19 appear homomorphic in both sexes of both species.
(2.24 MB TIF)

Figure S2 Karyograms of *C. inconstans*. (A) *C. inconstans* male. (B) *C. inconstans* female.
(6.62 MB TIF)

Table S1 Sex-linked *G. aculeatus* and *P. pungitius* microsatellite markers used for genotyping. For each marker, the *G. aculeatus* linkage group (LG) designation and position in the *G. aculeatus* genome assembly (http://www.ensembl.org/Gasterosteus_aculeatus/index.html) identified by BLAT are indicated. If a marker was also mapped in *G. wheatlandi* or *P. pungitius*, the LG designation in that species is also indicated. FP (failed PCR) indicates that PCR product was not obtained for a marker in a given species. NI (not informative) indicates that a PCR product was obtained in the species, but was not informative in a cross. NT (not tested) refers to markers for which sex linkage in a species could not be tested.
(0.08 MB DOC)

Table S2 Marker genotype-sex phenotype associations in *G. wheatlandi*. For each marker from *G. aculeatus* LG12 or LG19, the Kruskal-Wallis test statistic was used to determine whether there were significant differences in phenotype means between the four possible segregating genotypes “ac”, “ad”, “bc” and “bd”. For each marker, the mother was assigned genotype “ab” and the father was assigned genotype “cd”. The female sex phenotype was assigned a score of “0” and the male sex phenotype was assigned a score of “1”. The total number of individuals with a given marker genotype are indicated (*n*).
(0.05 MB DOC)

Table S3 Genome-wide microsatellite markers used for genotyping *C. inconstans* and *A. quadracus*.
(0.03 MB DOC)
