# Supplementary figures and images for: Turnover of Sex Chromosomes in the Stickleback Fishes (Gasterosteidae)
Source: PLoS Genet. 2009 Feb 20;5(2):e1000391. doi: 10.1371/journal.pgen.1000391 (PMC2638011; doi:10.1371/journal.pgen.1000391)

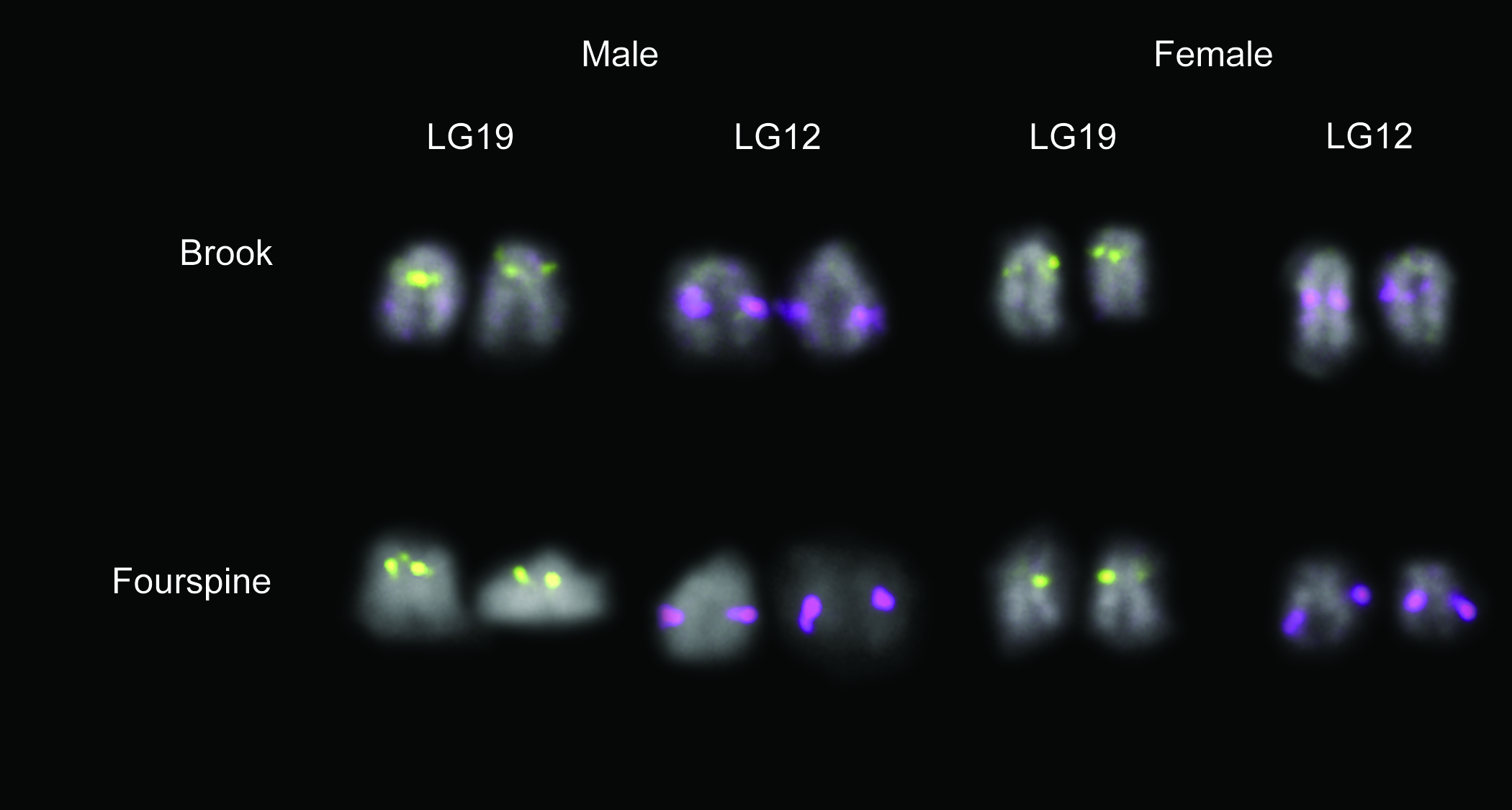

Supplement: Figure S1 — Fluorescence in situ hybridization analyses of LG12 and LG19 in C. inconstans and A. quadracus. The LG19 probe (CH213-180J08) is green, and the LG12 probe (CH213-140B10) is purple. For each sex of each species, only chromosomes hybridized by probe in a single metaphase spread are shown. LG12 and LG19 appear homomorphic in both sexes of both species. (2.24 MB TIF) [file pgen.1000391.s001.tif]

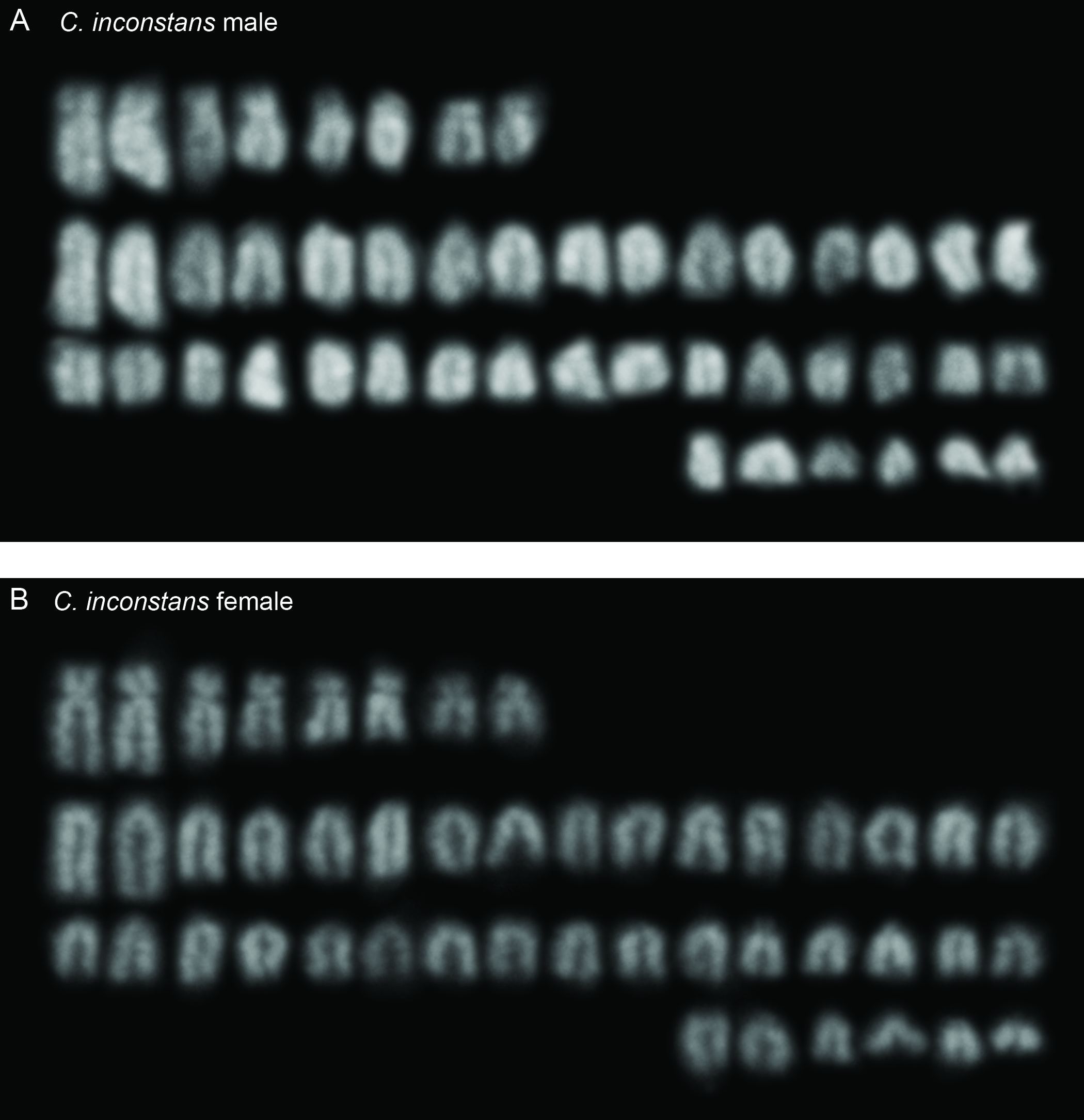

Supplement: Figure S2 — Karyograms of C. inconstans. (A) C. inconstans male. (B) C. inconstans female. (6.62 MB TIF) [file pgen.1000391.s002.tif]
