# Supplementary material for: Turnover of Sex Chromosomes in the Stickleback Fishes (Gasterosteidae)
Source: PLoS Genet. 2009 Feb 20;5(2):e1000391. doi: 10.1371/journal.pgen.1000391 (PMC2638011; doi:10.1371/journal.pgen.1000391)
Supplement: Table S1 — Sex-linked G. aculeatus and P. pungitius microsatellite markers used for genotyping. For each marker, the G. aculeatus linkage group (LG) designation and position in the G. aculeatus genome assembly (http://www.ensembl.org/Gasterosteus_aculeatus/index.html) identified by BLAT are indicated. If a marker was also mapped in G. wheatlandi or P. pungitius, the LG designation in that species is also indicated. FP (failed PCR) indicates that PCR product was not obtained for a marker in a given species. NI (not informative) indicates that a PCR product was obtained in the species, but was not informative in a cross. NT (not tested) refers to markers for which sex linkage in a species could not be tested. (0.08 MB DOC) [file pgen.1000391.s003.doc]

|  | *G. aculeatus* | | | *G. wheatlandi* | | *P. pungitius* | | *C. inconstans* | | *A. quadracus* | |
| --- | --- | --- | --- | --- | --- | --- | --- | --- | --- | --- | --- |
| Marker | LG | Position (Mb) | Sex-linked | LG | Sex-linked | LG | Sex-linked | PCR product | Sex-linked | PCR product | Sex-linked |
| *Pun99* | 12 | 5.576 | NT | 12/19 | yes | 12 | yes | FP | NT | yes | no |
| *Stn327* | 12 | 5.800 | no | 12/19 | yes | FP | NT | NI | NT | NI | NT |
| *Pun7* | 12 | 8.475 | no | NI | NT | 12 | yes | NI | NT | yes | no |
| *Stn287* | 12 | 9.516 | no | 12/19 | NT | 12 | yes | NI | NT | yes | no |
| *Stn276* | 12 | 9.516 | no | 12/19 | NT | 12 | yes | NI | NT | yes | no |
| *Stn144* | 12 | 11.037 | no | FP | NT | 12 | yes | NI | NT | yes | no |
| *Stn142* | 12 | 12.635 | no | 12/19 | yes | NI | NT | FP | NT | yes | no |
| *Pun2* | 12 | 12.276 | no | 12/19 | yes | 12 | yes | NI | NT | NI | NT |
| *Pun234* | 12 | 15.613 | NT | FP | NT | 12 | yes | yes | no | yes | no |
| *Stn186* | 19 | 1.942 | yes | NI | NT | 19 | no | yes | no | FP | NT |
| *Pun117* | 19 | 6.325 | yes | 12/19 | yes | 19 | no | FP | NT | yes | no |
| *Stn235* | 19 | 7.396 | yes | 12/19 | yes | NI | NT | FP | NT | FP | NT |
| *Stn194* | 19 | 11.787 | yes | 12/19 | yes | 19 | no | NI | NT | yes | no |
| *Pun268* | 19 | 13.170 | FP | FP | NT | 19 | no | yes | no | FP | NT |
| *Stn284* | 19 | 13.658 | yes | 12/19 | yes | NI | NT | NI | NT | FP | NT |
| *Stn168* | 19 | 13.736 | NI | NI | NT | 19 | no | yes | no | FP | NT |
| *Cyp19b* | 19 | 16.671 | yes | 12/19 | yes | 19 | no | yes | no | FP | NT |
