# Supplementary material for: Turnover of Sex Chromosomes in the Stickleback Fishes (Gasterosteidae)
Source: PLoS Genet. 2009 Feb 20;5(2):e1000391. doi: 10.1371/journal.pgen.1000391 (PMC2638011; doi:10.1371/journal.pgen.1000391)
Supplement: Table S2 — Marker genotype-sex phenotype associations in G. wheatlandi. For each marker from G. aculeatus LG12 or LG19, the Kruskal-Wallis test statistic was used to determine whether there were significant differences in phenotype means between the four possible segregating genotypes “ac”, “ad”, “bc” and “bd”. For each marker, the mother was assigned genotype “ab” and the father was assigned genotype “cd”. The female sex phenotype was assigned a score of “0” and the male sex phenotype was assigned a score of “1”. The total number of individuals with a given marker genotype are indicated (n). (0.05 MB DOC) [file pgen.1000391.s004.doc]

|  |  |  |  |  | | | |
| --- | --- | --- | --- | --- | --- | --- | --- |
|  |  |  |  | Phenotype means | | | |
| Marker | LG | Kruskal-Wallis | *p* | ac (*n*) | bc (*n*) | ad (*n*) | bd (*n*) |
| *Pun99* | 12 | 7.00 | <0.01 | 0.00 (4) | - | 1.00 (4) | - |
| *Stn327* | 12 | 76.00 | < 0.0001 | 0.00 (24) | 0.00 (16) | 1.00 (20) | 1.00 (17) |
| *Stn142* | 12 | 77.00 | < 0.0001 | 0.00 (21) | 0.00 (19) | 1.00 (24) | 1.00 (14) |
| *Pun2* | 12 | 43.00 | < 0.0001 | 0.00 (11) | 0.00 (14) | 1.00 (10) | 1.00 (9) |
| *Pun117* | 19 | 75.00 | < 0.0001 | 0.00 (23) | 0.00 (17) | 1.00 (21) | 1.00 (15) |
| *Stn235* | 19 | 59.00 | < 0.0001 | 0.00 (16) | 0.00 (16) | 1.00 (16) | 1.00 (12) |
| *Stn194* | 19 | 61.00 | < 0.0001 | 0.00 (13) | 0.00 (17) | 1.00 (21) | 1.00 (11) |
| *Stn284* | 19 | 79.00 | < 0.0001 | 0.00 (18) | 0.00 (23) | 1.00 (24) | 1.00 (15) |
| *Cyp19b* | 19 | 65.00 | < 0.0001 | 0.00 (13) | 0.00 (20) | 1.00 (20) | 1.00 (13) |
|  |  |  |  |  |  |  |  |
