# Supplementary material for: Turnover of Sex Chromosomes in the Stickleback Fishes (Gasterosteidae)
Source: PLoS Genet. 2009 Feb 20;5(2):e1000391. doi: 10.1371/journal.pgen.1000391 (PMC2638011; doi:10.1371/journal.pgen.1000391)
Supplement: Table S3 — Genome-wide microsatellite markers used for genotyping C. inconstans and A. quadracus. (0.03 MB DOC) [file pgen.1000391.s005.doc]

|  | |  | | |  | | |
| --- | --- | --- | --- | --- | --- | --- | --- |
|  | | *C. inconstans* | | | *A. quadracus* | | |
| Species | *n* | PCR product | Informative | Sex-linked | PCR product | Informative | Sex-linked |
| *G. aculeatus* | 576 | 225 (39.1%) | 66 (11.5%) | 0 (0%) | 135 (23.4%) | 47 (8.2%) | 0 (0%) |
| *P. pungitius* | 162 | 86 (53.1%) | 43 (26.5%) | 0 (0%) | 51 (31.5%) | 26 (16.1%) | 0 (0%) |
